# Performance comparison of bio-inspired and learning-based clustering analysis with machine learning techniques for classification of EEG signals

**DOI:** 10.3389/frai.2023.1156269

**Published:** 2023-06-21

**Authors:** Sunil Kumar Prabhakar, Dong-Ok Won

**Affiliations:** Department of Artificial Intelligence Convergence, Hallym University, Chuncheon, Republic of Korea

**Keywords:** epilepsy, EEG, K-means clusters, fuzzy C-means clusters, Cuckoo search clusters, Firefly clusters, Dragonfly clusters

## Abstract

A comprehensive analysis of an automated system for epileptic seizure detection is explained in this work. When a seizure occurs, it is quite difficult to differentiate the non-stationary patterns from the discharges occurring in a rhythmic manner. The proposed approach deals with it efficiently by clustering it initially for the sake of feature extraction by using six different techniques categorized under two different methods, e.g., bio-inspired clustering and learning-based clustering. Learning-based clustering includes K-means clusters and Fuzzy C-means (FCM) clusters, while bio-inspired clusters include Cuckoo search clusters, Dragonfly clusters, Firefly clusters, and Modified Firefly clusters. Clustered values were then classified with 10 suitable classifiers, and after the performance comparison analysis of the EEG time series, the results proved that this methodology flow achieved a good performance index and a high classification accuracy. A comparatively higher classification accuracy of 99.48% was achieved when Cuckoo search clusters were utilized with linear support vector machines (SVM) for epilepsy detection. A high classification accuracy of 98.96% was obtained when K-means clusters were classified with a naive Bayesian classifier (NBC) and Linear SVM, and similar results were obtained when FCM clusters were classified with Decision Trees yielding the same values. The comparatively lowest classification accuracy, at 75.5%, was obtained when Dragonfly clusters were classified with the K-nearest neighbor (KNN) classifier, and the second lowest classification accuracy of 75.75% was obtained when Firefly clusters were classified with NBC.

## 1. Introduction

Due to the excessive and abnormal electrical discharges in the brain cells, seizures are produced, which occur suddenly and are quite uncontrollable, making epilepsy a chronic neurological disorder (Gotman, 1982). To monitor the activities of the brain, electroencephalography (EEG) is widely used as it provides a huge amount of both physiological and pathological information, thereby proving its validity for an effective diagnosis (Kim et al., 2014; Jukic et al., 2020). The application of EEG plays a vital role in almost all areas of biomedical engineering, ranging from seizure classification (Rajaguru and Prabhakar, 2016), Alzheimer's disease diagnosis (Zhu et al., 2016), subject-dependent classification in Brain-Computer Interface (BCI) (Lee et al., 2015), classification of steady-state visual evoked potentials (Won et al., 2015), efficient analysis of Event-Related Potentials (ERP)-based BCI (Yeom et al., 2014), analysis of deep neural network modeling under an ambulatory environment (Kwak et al., 2017), dementia classification (Jeong, 2004), and iris recognition (Adamovic et al., 2020). Visual analysis of EEG recordings, even by expert neurologists, is very difficult and time-consuming. The over-reliance and subjective judgments of visual encephalographs may result in different diagnoses of the EEG segment (Kumar et al., 2014). As the EEG recordings have a very high margin of noise, separating seizures from artifacts is difficult as they have a similar time-frequency pattern. A plethora of machine-learning algorithms have been proposed for seizure detection and classification (Magosso et al., 2009). Generally, in the initial stages, preprocessing and feature extraction are performed and then classified with supervised or unsupervised classification models. To remove the major artifacts caused by eye blinking, slight body movement, and random muscle activity, preprocessing is performed, which helps in the extraction of the most discriminative features in the time domain (Bulthoff et al., 2003). Thus, studies on seizure detection using epileptic EEG databases are of great significance.

**Figure 1:**
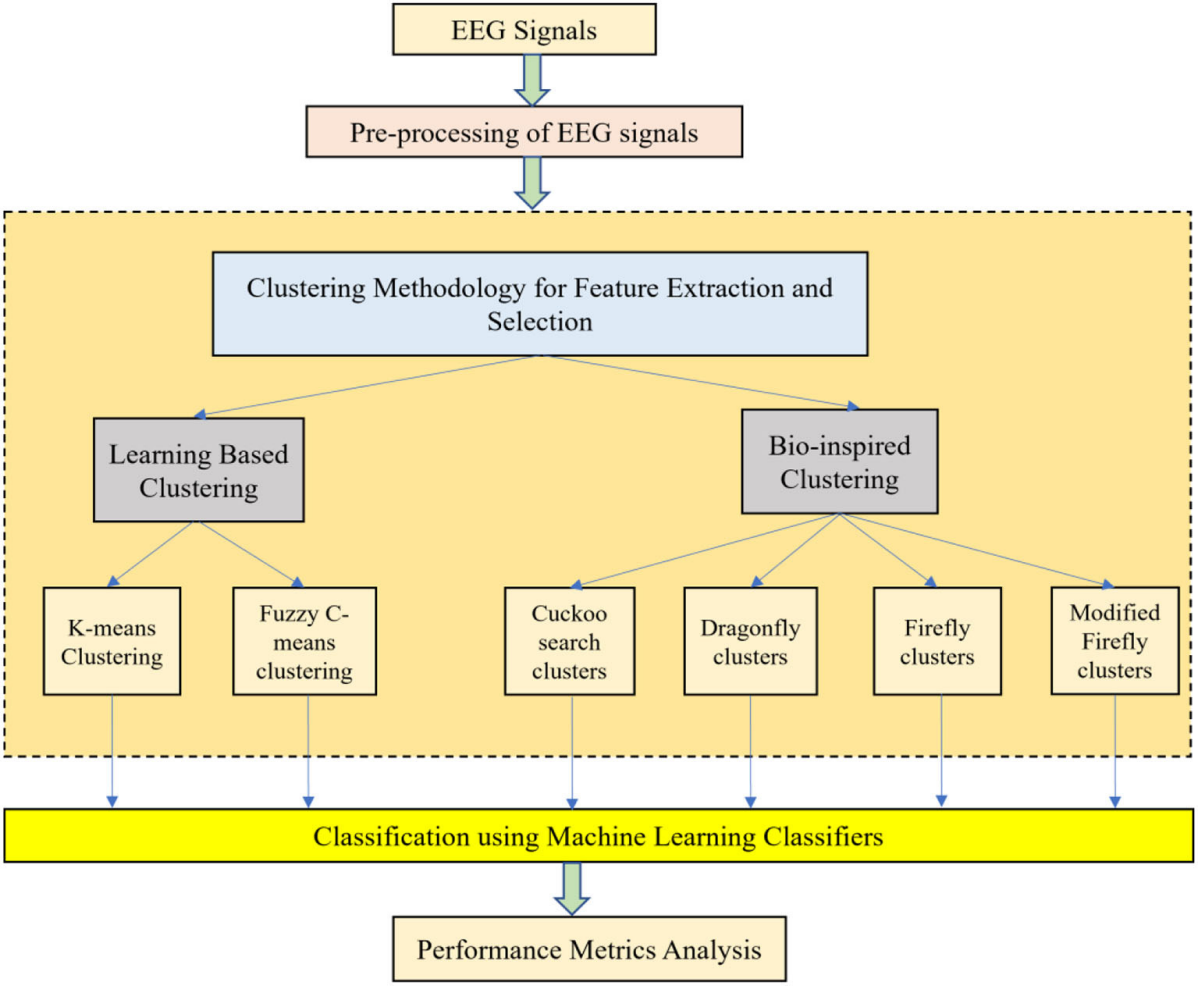
Simplified process flow of the methodology.

With the help of Artificial Neural Networks (ANN), a classification accuracy of 100% between normal and epileptic EEG signals has been achieved using Approximate Entropy (ApEn) by Srinivasan et al. (2007). For the automated detection of epileptic seizures from EEG signals, many researchers have proposed different feature extraction methodologies, as explained in (Tawfik et al., 2016). With the help of both Bayesian Linear Discriminant Analysis (BLDA) and lacunarity analysis, an algorithm for intracranial EEG seizure detection was proposed by Zhou et al. with a sensitivity of about 96.25% (Zhou et al., 2013). A good review of different entropy methods to differentiate normal, ictal, and interictal EEG signals with seven different classifiers was done by Acharya et al. (2012) where a high classification accuracy of 98.1% was reported. Compressive sensing techniques using Sample entropy, permutation entropy, and Hurst index for differentiating and detecting inter-ictal, pre-ictal, and ictal-based epileptic seizures with four different classifiers were done by Zeng et al. (2016), and a high classification accuracy of 76.7% was obtained. A 13-layer deep Convolutional Neural Network (CNN) was employed to detect normal, pre-ictal, and seizure classes in (Acharya et al., 2018) and reported accuracy, specificity, and sensitivity of 88.67, 90, and 95%, respectively. Automated seizure detection from EEG signals using a deep Convolutional Neural Networks (CNN)-based technique yielding a high classification accuracy of 97.5% was performed in (Zhou et al., 2018). SVM classifiers are one of the most versatile pattern recognition techniques used in epileptic seizure detection due to their most promising ability for generalization (Moghim and Corne, 2014). Constructive Genetic Programming (CGP) was used for classification after feature extraction by decomposing EEG signals using Empirical Mode Decomposition (EMD) by Bhardwaj et al. (2016), and for different validation schemes, the classification accuracy results were reported as ranging from 97 to 100%. ANNs were incorporated with multivariate EMD to classify ictal vs. non-ictal signals with the help of a standard SVM classifier (Riaz et al., 2016). Frequency domain features were reduced using non-linear dimension techniques, and the seizures were then classified using a K-nearest neighbor (KNN) classifier, which reported a classification accuracy of 98.40% (Rivero et al., 2011). Smoothed pseudo-Wigner-Ville distribution was incorporated with ANNs for seizure classification, as proved in (Tzallas and Tsipouras, 2007), reporting an overall accuracy of 97.72 to 100%. Wavelet transforms also gave good results in the analysis of epilepsy detection studies, as shown in (Chen et al., 2017), where a perfect classification rate of 100% was obtained with all kinds of wavelet filters. Usage of techniques like tunable Q-factor wavelet transform with bootstrapping methodology gave a 100% classification accuracy, especially for A-E seizure classification problems (Haasan and Siuly, 2016), multi-wavelet-based approximate entropy (ApEn), with ANN again reporting a 100% classification accuracy (Kumar et al., 2014), and complex-valued neural network transform with K-fold cross-validation methodology also reporting results from 99 to 100% classification accuracy (Peker and Sen, 2016) are some of the predominantly used methodologies for epilepsy classification. A sparse autoencoder with a swarm-based deep learning technique (Prabhakar and Lee, 2022a), sparse representation-based robust hybrid feature extraction models with transfer and deep learning (Prabhakar and Lee, 2022b), sparse modeling with an ensemble and nature-inclined classification (Prabhakar and Lee, 2022c), and end-to-end deep neural network models (Zhao et al., 2020) were also done for epilepsy classification from EEG signals. Other important studies deal with the application of matrix determinants with machine learning classifiers (Raghu et al., 2019), local mean decomposition (LMD) with genetic algorithm-supported SVM (GA-SVM) (Zhang and Chen, 2017), discrete wavelet transform (DWT) with SVM (Chen et al., 2017), and linear least squares preprocessing with SVM (Zamir, 2016) for the classification of epilepsy from EEG signals. Other prominent studies include the usage of orthogonal wavelet analysis with linear SVM (Sharma et al., 2018), weighted complex networks with SVM (Diykh et al., 2017), Fourier transform with neural networks (Samiee et al., 2015), and binary pattern generation with Bayesian networks (Kaya et al., 2014) for the classification of epilepsy from EEG signals. In this study, the A-E classification problem of the Bonn data set was thoroughly studied through feature extraction, initially through clustering techniques, followed by categorization using appropriate classifiers for epilepsy from EEG signals. The organization of the work is as follows: Section 2 presents the materials and methods, followed by feature extraction through the clustering method in Section 3. The classification process is discussed in Section 4. It is followed by results and discussion in Section 5 and concludes in Section 6. The block diagram of the work is shown in Figure 1. The EEG signals taken from the Bonn data set were first preprocessed using the Independent Component Analysis (ICA) method and then clustered using two methodologies. Learning-based clustering includes k-means clustering and FCM clustering, while bio-inspired clustering involves cuckoo search clustering, dragonfly clustering, firefly clustering, and modified firefly clustering. The most important works in this field with interesting results in the last 6 years are shown in Table 1.

**Table 1:** A few famous and important epilepsy classification studies that have used the Bonn data set in the past.

**Year**	**References**	**Classification type**	**Technique adopted**
2022	Prabhakar and Lee (2022a)	A-E, B-E, C-E, D-E, AB-E, CD-E, AC-E, ACD-E, ABCD-E, BCD-E	Sparse autoencoder with swarm-based deep learning and reinforcement-based Q-learning
2022	Prabhakar and Lee (2022b)	B-E, AB-E, D-E, CD-E, A-C-E, AB-CD-E	Sparse representation-based robust hybrid feature extraction models with transfer and deep learning
2022	Prabhakar and Lee (2022c)	A-E, B-E, C-E, D-E, AB-E	Sparse modeling with ensemble- and nature-inclined classification
2020	Zhao et al. (2020)	A-E, B-E, C-E, D-E, AB-E, CD-E, ABCD-E	End-to-end deep neural network model
2019	Raghu et al. (2019)	A-E, B-E, C-E, D-E, AB-E, AC-E, CD-E, ACD-E, ABCD-E, A-C-E, AB-CD-E	SVM, KNN, Multi-layer perceptron (MLP), CNN
2018	Zhou et al. (2018)	A-E, A-C	CNN
2017	Zhang and Chen (2017)	A-E, D-E, ABCD-E, A-D-E, A-B-C-D-E	Local mean decomposition (LMD)-based hybrid features and GA-SVM
2017	Chen et al. (2017)	A-E, AB-E, CD-E, A-CDE, ACD-E, AB-CDE, ABCD-E	Discrete wavelet transform (DWT) and SVM
2016	Bhardwaj et al. (2016)	A-E, ACD-E, ABCD-E, AB-CD-E, A-B-C-D-E	EMD and CGP
2016	Zamir (2016)	A-E, B-E, ABCD-E, ACD-E	Linear least squares pre-processing and library SVM

## 2. Materials and methods

The data used in this study is a publicly available data from the Epilepsy Research Center at Bonn University in Germany (Andrzejak et al., 2001). It consists of five subsets indicated as A-E. Each subset has 100 single-channel segments, and each segment comprises an EEG signal of 23.6 s duration with a sampling frequency of ~173.61 Hz. The EEG signals present in this public data set were obtained from a 128-channel amplification system with an average reference electrode. In this study, the A-E classification type was studied in detail using clustering techniques and machine learning procedures. Further details about the EEG data set are given in Table 2. As the presence of abnormalities in the EEG signals is quite high due to its non-stationary and non-linearity properties, clustering is an effective methodology to extract useful features.

**Table 2:** Bonn data set description details.

**Subset**	**A**	**B**	**C**	**D**	**E**
Subjects	Five healthy volunteers		Five epileptic patients		
Subject state	Eyes open	Eyes closed	Inter-ictal		Ictal
Recorded period	Normal		Seizure-free intervals		Seizure activity
Electrode types	Non-invasive surfaces		Intracranial		
Placement of electrodes	International 10–20 standard system		From hippocampal formation	Epileptogenic zone	All epileptic seizure areas

## 3. Clustering techniques

Clustering is basically the primary task of grouping or assimilating a set of specific objects in such a manner that the objects in the same group are very similar to each other. It is a vital task in exploratory data mining and is commonly used for all kinds of statistical data analysis in the fields of machine learning, pattern recognition, data analysis, and more (Zhao et al., 2020). Cluster analysis cannot be limited to one specific algorithm. Different algorithms can be used for cluster analyses, which differ significantly in their basic understanding of the constitution of the cluster. The formulation of a clustering problem is generally done as a multi-objective optimization problem, as most popular clusters include groups with dense areas of data space and small distances between the cluster members.

When working with large amounts of data, classification is generally difficult. Therefore, data reduction or data partitioning methods are quite useful for this purpose. After the basic pre-processing of EEG signals using Independent Component Analysis (ICA), data reduction is achieved by various methods like feature extraction, feature selection, and clustering techniques. Clustering is an unsupervised method used for data reduction. Selecting the number of clusters that represent the original data is important for the classification process. This will reduce the burden on the classifier and thereby increase classification accuracy. Clustering (unsupervised methods) can be defined as the process of organizing data into groups whose members are similar in some way. Without using training data, they normally work as an optimizer. To compensate for the lack of training data, this alternative method efficiently analyzes the partitioned data, thereby characterizing the properties of each class in a very distinctive way. The EEG signals are clustered as K-means clusters, FCM clusters, Cuckoo search clusters, Dragonfly clusters, Firefly clusters, and modified Firefly clusters.

### 3.1. K-means clustering

The problem of clustering a set of m objects *J* = {1, ..., *m*} objects into *K* clusters is initially considered in K-means clustering (Kanungo et al., 2004). For every subject *j* ∈ *J*, we have a set of *n*features [*z*_*ji*_ : *i* ∈ *I*], where *z*_*ji*_ explains the *i*^*th*^ features of the object *j* in a quantitative manner. Assuming $z_{j}={\left(z_{j1},...,z_{jn}\right)}^{T}$is the feature vector of the object *j* and *Z* = (*z*_1_, ..., *z*_*m*_) is the data set or the feature matrix, this clustering task is nothing but a reformulation as an optimization problem, thereby minimizing the clustering objective function as follows:


(1) $$\begin{array}{l} \min J\left(A,B\right)=\overset{K}{\underset{k=1}{\sum }}\underset{j=J}{\sum }{a_{jk}||z_{j}-b_{k}||}_{q}^{q} \end{array}$$

Under the following condition $\overset{K}{\underset{k=1}{\sum }}a_{jk}=1,a_{jk}\in \left\{0,1\right\},\forall_{j}\in J,k=1,...,K$, where *q* = 1, 2. For *k* = 1, ..., *K*, $b_{k}\in {ℜ}^{m}$ is the *k*^*th*^ cluster prototype, and for any *j* ∈ *J*, *a*_*jk*_ denotes whether the object *j* belongs to the *k*^*th*^ cluster.

To solve the clustering problems for *q* = 1 and *q* = 2, K-median and K-means are quite effective algorithms. The cluster prototype matrix $B=\left[b_{1},...,b_{K}\right]\in {ℜ}^{n\times K}$ and the membership matrix $A=\left[a_{1},...,a_{m}\right]\in {ℜ}^{K\times m}$, where $b_{j}={\left(b_{j1},...,b_{jm}\right)}^{T}$ and $a_{j}={\left(a_{j1},...,a_{jK}\right)}^{T}$. In an iterative manner, the clustering problem is solved by the Algorithm 1 as follows:

#### 3.1.1. Algorithm 1: K-means clustering

Step 1: The iteration index *t* = 0 is set first, and then a random selection of *K*different objects is depicted as the initial cluster prototype and is represented as:


(2) $$\begin{array}{l} \left\{b_{k}^{t}\;:\;k=1,...,K\right\} \end{array}$$

Step 2: Assuming that *t* = *t* + 1, then the membership matrix *A*^*t*^ is updated by fixing the cluster prototype matrix *B*^*t*−1^. For any *j* ∈ *J*, the random selection of $k^{*}\in \arg\min\left\{{\left||z_{j}-b_{k}^{t-1}|\right|}_{q}:k=1,...,K\right\}$ and set $a_{jk^{*}}^{t}=1$ and for any *k* ≠ K^*^, set $a_{jk}^{t}=0$

Step 3: The cluster prototype matrix *B*^*t*^ is updated by means of fixing the membership matrix *A*^*t*^. When *q* = 1, for any *k* = 1, ..., *K* and *i* ∈ *I*, set $b_{k_{i}}^{t}$as the median of the *i*^*th*^ feature values of these objects in the cluster *k*, when *q* = 2, for any *k* = 1, ..., *K*, set $b_{k}^{t}$ as the centroid of these objects in the cluster *k*, (i.e.,):


(3) $$\begin{array}{l} b_{k}^{t}=\left(1/\Sigma_{j\in J}a_{jk}\right)\Sigma_{j\in J}a_{jk}z_{j} \end{array}$$

Step 4: For any *j* ∈ *J* and *k* = 1, ..., *K*, we have $a_{jk}^{t}=a_{jk}^{t-1}$, then the process is stopped and returned to *A* and *B*; otherwise we return to step 2 of Algorithm 1.

### 3.2. Fuzzy C-means clustering

While dealing with fuzzy C-means clustering, *Y* = {*y*_1_, *y*_2_, ..., *y*_*n*_} is a finite data set considered for our analysis where *y*_*i*_ = (*y*_*i*1_, *y*_*i*2, ..._,*y*_*ig*_) is a *g*-dimensional object and *y*_*ig*_ is the *g*^*th*^ property of the *i*^*th*^ object. *K* = {*K*_1_, *K*_2_, ..., *K*_*k*_} denotes ′*k*′ clusters. *W* = {*w*_1_, *w*_2_, ..., *w*_*k*_} represents *k*1-dimensional cluster centroids, where *w*_*i*_ = (*w*_*i*1_, *w*_*i*2_, ..., *w*_*ig*_). *Z* = (*z*_*iq*_)_(*n*×*k*)_ is a fuzzy partition matrix and *z*_*iq*_ denotes the degree of membership of the *i*^*th*^ object in the *q*^*th*^ cluster where $\sum_{q=1}^{k}z_{iq}=1,\forall_{i}=1,...,n$. The quadratic sum of weighted clusters to the cluster centroid from the samples in each cluster is the objective function and is denoted as:


(4) $$\begin{array}{l} H_{m}\left(Z,W\right)=\overset{k}{\underset{q=1}{\sum }}\overset{n}{\underset{i=1}{\sum }}z_{iq}^{p}d_{iq}^{2} \end{array}$$

where *d*_*iq*_ = ||*y*_*i*_ − *w*_*q*_|| depicts the Euclidean distance between the *i*^*th*^ object and the *q*^*th*^cluster point. (*p* ∈ [1, ∞]) denotes a fuzziness index that helps in fuzzy membership control (Zhang et al., 2016). The resulting membership will be fuzzier if the value of *p* is higher. Based on the clustering idea, the appropriate fuzzy partition matrix *Z* and cluster centroid *W* are obtained (to minimize the objective function *H*_*m*_). Depending on the Lagrange multiplier technique *Z* and *W* it is calculated as:


(5) $$\begin{array}{l} z_{iq}=\frac{1}{\sum_{h=1}^{k}{\left(d_{iq}/d_{hq}\right)}^{{}^{2}{/}_{\left(p-1\right)}}}\\ w_{q}=\frac{\sum_{i=1}^{n}z_{iq}^{p}y_{i}}{\sum_{i=1}^{n}z_{iq}^{p}} \end{array}$$

The FCM algorithm is implemented by means of minimizing the objective function *H*_*p*_ with the updates of *Z* and *W*. The steps are as follows as mentioned in Algorithm 2.

#### 3.2.1. Algorithm 2: fuzzy C-means clustering

1. The initial value of the total number of clusters ′*k*′, fuzziness index *p*, threshold ξ, and maximum iterations *I*_max_ is assigned.
2. The fuzzy partition *Z*^(0)^ is randomly initialized based on the degree of membership.
3. The *k* cluster centroid *W*^(*t*)^ is calculated at the t-step.
4. The objective function $H_{p}^{\left(t\right)}$ is calculated. If $\left|H_{p}^{\left(t\right)}-H_{p}^{\left(t-1\right)}\right|<\xi$ or *t* > *I*_max_, we stop or continue to the next step.
5. *Z*^(*t*+1)^ is computed according to step 2 of this algorithm and then we proceed to step 3 of Algorithm 2.

Thus, it is quite a simple algorithm and its simplicity can be extended because of its quick convergence. Then, the conventional method to assess the optimal number of FCM clusters consists of the following steps.

Step 1: The search range [*k*_min_, *k*_max_] must be fed or input; generally, *k*_min_ = 2 and $k_{\max}=\left[\sqrt{n}\right]$

Step 2: For every integer *qn* ∈ [*k*_min_, *k*_max_]

Step 3: FCM is executed

Step 4: The clustering validity index is calculated

Step 5: The value of the clustering validity index is compared

Step 6: *k*_*opt*_ is obtained, which gives the clustering result.

### 3.3. Cuckoo clustering

Cuckoo search is a bio-inspired algorithm in which some species of cuckoo birds with obligate brood parasitism lay their eggs in the nests of some other bird species (Abd Elazim and Ali, 2016). Using the general rules, the Cuckoo search algorithm can be described as follows.

1. Each cuckoo lays one egg at a time and then it deposits it in a randomly selected nest.
2. The best nests with very good quality eggs will be passed on to the next generation.
3. The total number of available host nests is fixed. The host bird discovers the egg laid by a cuckoo with a probability of *P*_*a*_ ∈ [0, 1]. In such a case, the host bird can abandon the nest and build a new one, or easily get rid of the egg. The method is explained as follows in Algorithm 3.

**Algorithm 3:** Cuckoo clustering.

**Objective function:** *f*(*y*) = *y* = (*y*_1_, *y*_2_, ..., *y*_*m*_)
**Generate:** an initial population of *q*host nests
**While** (*t*<Maximum Generation) or (Criteria to Stop)
Select a random cuckoo (say, *j*) and solution replacement by performing Levy flights
Fitness *F*_*i*_ evaluation
Randomly choose a nest among *m*(say, *i*)
**if** *F*_*j*_ < *F*_*i*_ then
Replace *i* by the new solution
**End if**
A fraction *p*_*a*_ of worse nests are abandoned and new ones are built
The best ones are retained and kept
Ranking of nests is done to find the current best
The current best solution is passed to the next generation
**End while**

### 3.4. Dragonfly clustering

Dragonflies are assumed to be little predators that naturally hunt other little bugs (Sree Ranjini and Murugan, 2017). Even before the marine bugs and little fishes originated, fairy dragonflies came into existence. The unusual swarming behavior is the most fascinating certainty about dragonflies. The two basic swarming methods are the same techniques of streamlining using metaheuristics, namely investigation and misuse. Some default steps like separation/split, alignment/join, cohesion, and attraction to a food source are common in this algorithm and are explained in Algorithm 4.

#### 3.4.1. Algorithm 4: dragonfly clustering

Step 1: Dragonfly initialization:

The population of dragonflies is initialized first and expressed as *Q*_*j*_ and its representation is done as:


(6) $$\begin{array}{l} H_{e1}=\left[H_{e_{1},}H_{e_{2},}H_{e_{3}},...,H_{e_{n}}\right] \end{array}$$

The four important phases in Dragonfly initialization are as follows.

- Separation phase: To neglect the static collision of the people from various people in the neighborhood, the separation process is utilized and expressed as:
  (7) $$\begin{array}{l} Sep_{je}=\overset{M}{\underset{l=1}{\sum }}H_{e_{j}}-H_{e_{l}} \end{array}$$
  where *Sep*_*e*_*j*__ denotes the separation of the *j*^*th*^ individual, *H*_*e*_ denotes the present position of the individual, and *H*_*e*_*l*__ is the position of the *l*^*th*^ individual, and *M* denotes the total number of individuals in an adjacent nearer area present in the search space.
- Alignment phase: This happens depending on the velocity matching of each individual to other respective individuals in the neighborhood. It is calculated as:
  (8) $$\begin{array}{l} Ali_{j}=\frac{\sum_{l=1}^{M}E_{l}}{M} \end{array}$$
  where *Ali*_*j*_ denotes the alignment of the *j*^*th*^ neighboring individual, is the velocity of the *l*^*th*^ individual, and *M* denotes the total number of neighboring individuals in the entire search space.
- Cohesion phase: The dependency of the individuals to move toward the center of the mass in the neighborhood is called cohesion. The cohesion *Co*_*j*_ for the *j*^*th*^ individual is calculated as:
  (9) $$\begin{array}{l} Co_{j}=\frac{\sum_{l=1}^{M}H_{e_{l}}}{M}-H_{e} \end{array}$$
- Progressing toward a food source phase: Among the dragonflies, this is the attractive phase toward a particular food source, and the outward distance of each dragonfly is expressed as:
  (10) $$\begin{array}{l} Food_{j}=H_{e}^{+}-H_{e}^{-} \end{array}$$
  (11) $$\begin{array}{l} Ene_{j}=Q^{-}+D \end{array}$$
  where $H_{e}^{-}$ represents the position of the enemy and $H_{e}^{+}$ denotes the position of the food source.
  Step 2: Update process

(12) $$\begin{array}{l} \Delta Feature\text{\_}set=\left(v_{s}Sep_{j}+v_{a}Ali_{j}+v_{c}Co_{j}+v_{h}Food_{j}+v_{e}Ene_{j}\right)\\ \;+v\Delta Feature_{t} \end{array}$$

Two vectors, (Δ*Feature*) and position (*Feature*) vector are considered to update the position of artificial dragonflies in the search space, *v*_*s*_, *v*_*a*_, where it represents the weights of the technique, such as detachment, arrangement, and union. From the result of the calculation, the advanced positions are obtained, and thus, after checking the score estimates, additional information can be extracted.

### 3.5. Firefly clustering

It is a bio-inspired metaheuristic algorithm predominantly used for solving optimization problems. It is based on and observed in the flashing behavior of fireflies at night (Yang, 2010). The three main rules used in the construction of this algorithm are that, first, fireflies are unisexual in nature. Second, the brightness of the firefly is understood from the objective function. Third, there is a direct proportional relationship between attractiveness and brightness. A firefly generally moves toward the brighter one, and if there is no brighter one, then there is random movement. It is a well-known fact that there is an inversely proportional relationship between the intensity of light and the square of the distance, for instance, *q* from the source. Moreover, when light passes through a particular medium with a light absorption coefficient of λ, the intensity of light *J* varies with distance as follows:


(13) $$\begin{array}{l} J\left(q\right)=J_{0}e^{-\lambda q} \end{array}$$

where *J*_0_ denotes the intensity at the starting point. It can be combined as:


(14) $$\begin{array}{l} J\left(q\right)=J_{0}e^{-\lambda q^{2}} \end{array}$$

Computing *e*^−λ*q*^2^^ is slightly difficult, but computing $\frac{1}{1+\lambda q^{2}}$ is comparatively easier. Therefore, the calculation of the intensity is done as follows:


(15) $$\begin{array}{l} J\left(q\right)=\frac{J_{0}}{1+\lambda q^{2}} \end{array}$$

The definition of firefly attractiveness is expressed as follows:


(16) $$\begin{array}{l} B\left(q\right)=\frac{B_{0}}{1+\lambda q^{2}} \end{array}$$

where *B*_0_ denotes the attractiveness of *q* = 0. If a firefly situated at $y^{\prime }=\left(y_{1}^{\prime },y_{2}^{\prime },...,y_{n}\right)$ is brighter than another firefly situated at *y* = (*y*_1_, *y*_2_, ..., *y*_*n*_), then the firefly located at *y* moves toward *y*′. The update of the firefly located at *y* is expressed as:


(17) $$\begin{array}{l} y:=y+A_{0}e^{-\lambda q^{2}}\left(y^{\prime }-y\right)+\alpha \varepsilon \end{array}$$

αε denotes the randomization term with α represented as the randomization parameter denoted within the range of 0 to 1, i.e., 0 ≤ α ≤ 1, and ε denotes the random vector numbers. The second term is because of the attractiveness of *y* toward *y*′. For the sake of practicality, *A*_0_ can be assumed to be 1; *A*_0_ = 1.

The summary of the algorithm is given in Algorithm 5 as follows.

#### 3.5.1. Algorithm 5: firefly clustering

1. A random solution set {*y*_1_, *y*_2_, ..., *y*_*k*_} is generated.
2. The intensity for each solution member is computed as {*J*_1_, *J*_2_, ..., *J*_*k*_}.
3. Each firefly ′*j*′ moves toward other bright fireflies. If there are no bright fireflies, then each firefly moves in a random manner.
4. The solution set is updated.
5. If the termination criteria are fulfilled, then we terminate, otherwise, we go to step 2 of this algorithm.

### 3.6. Modified firefly clustering

The firefly with the current best global solution is the brightest. The random movement of this brightest firefly may decrease its brightness depending on the direction. Therefore, in that particular direction, it leads to the performance degradation of this algorithm. If the brightest firefly only moves in a direction that increases its brightness, then the algorithm can perform better. The modification made here is implemented in (Tilahun and Ong, 2012), and the idea is incorporated into our work as follows. To assess the direction of the brightest firefly, ′*s*′ unit vectors are randomly generated, e.g., *v*_1_, *v*_2_, ..., *v*_*s*_. Among the randomly generated ′*s*′ directions, a direction *V* is chosen in such a way that the brightness of the brightest firefly always increases when the firefly is in that particular direction. Therefore, the movement of the brightest firefly can be expressed as; *y*: = *y* + α*V*, where α denotes the random step length.

The brightest firefly will stay in its current position if such a direction does not exist in any of the randomly generated solutions. Moreover, rather than assuming $B_{0}^{j}=1$ for each firefly *j*, it is a good idea to assign some attractiveness that is dependent on the firefly intensity, which is in turn highly dependent on the objective function. One way to do this is to assign the ratio of the intensities of the fireflies. If a firefly *j* situated at *y*′ is brighter than a firefly *i* located at *y*, then the firefly located at *y* moves toward the firefly *j*, and *B*_0_ is expressed as:


(18) $$\begin{array}{l} B_{0}=\frac{J_{0}^{\prime }}{J_{0}} \end{array}$$

where $J_{0}^{\prime }$ is the intensity at *q* = 0 for firefly *j*, *J*_0_ is the intensity at *q* = 0 for firefly *i* and *J*_0_ ≠ 0. For convenience, we reject the singularity case when *J*_0_ = 0, *B*_0_ is expressed as $e^{J_{0}^{\prime }-J_{0}}$. If we consider $B_{0}=J_{0}^{\prime }$ and when the intensity is large, then the movement of the firefly *i* toward *j* is very long. However, *B*_0_ can be adjusted depending on the solution space. Thus, the solution space here should be directly proportional to the intensity at the source $J_{0}^{\prime }$.

The main reason for the clustering in this work is the data reduction through the process of optimization, as stated in the above algorithms. The clusters are validated by two parameters, compactness and separability. In this study, the compactness of the clusters is analyzed by the scatter plots among the classes of EEG data. When it comes to separability, these particular types of indices are used to differentiate between two clusters. The distance between the two cluster centroids is a commonly used measure of separability. This measure is easy to compute and can detect even hyperspherical clusters. Three types of entropy, e.g., Approximate Entropy, Shannon Entropy, and Sample Entropy were used to identify the separability of the clusters among the classes of the EEG data set (A-E), which is shown in Table 3.

**Table 3:** Average entropy values.

**Clusters**	**Approximate entropy**		**Shannon entropy**		**Sample entropy**	
EEG sets	A	E	A	E	A	E
K-means	1.37	1.19	1.46	1.24	5.429	6.15
FCM	1.42	1.15	1.58	1.27	5.23	6.34
Cuckoo	1.86	1.07	1.92	0.864	4.1774	6.82
Dragonfly	1.79	1.246	1.83	1.12	4.637	5.96
Firefly	1.65	1.058	1.75	1.034	5.407	6.12
Modified Firefly	1.39	1.217	1.86	1.17	4.8019	6.45
Average	1.58	1.155	1.733	1.16	4.95	6.32

**Figure 2:**
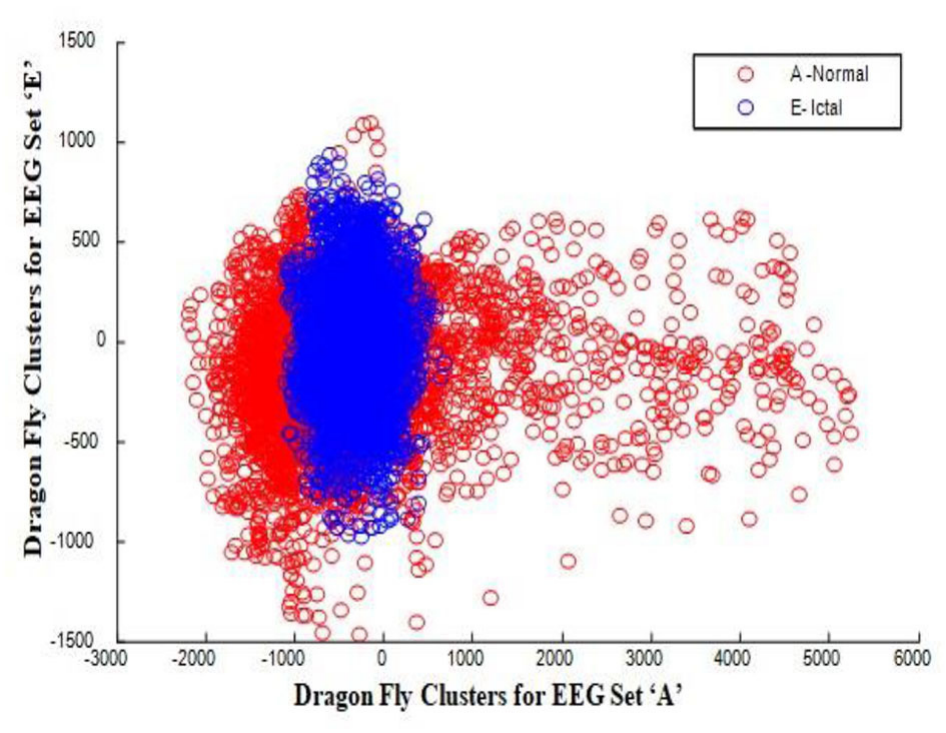
Dragonfly clusters for EEG sets A vs. E.

**Figure 3:**
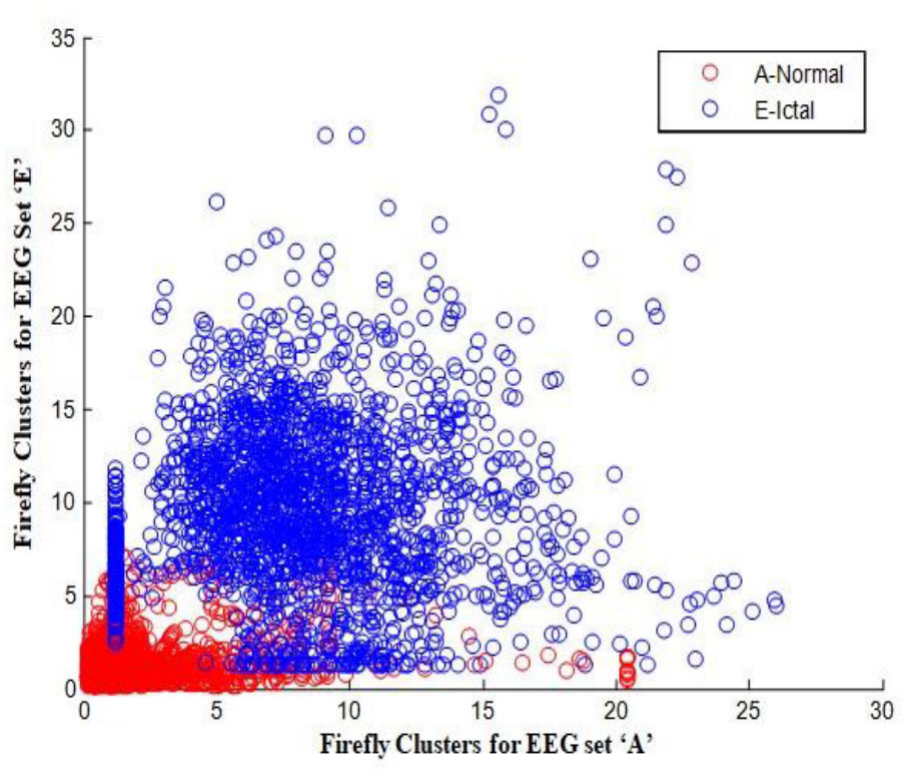
Firefly clusters for EEG sets A vs. E.

**Figure 4:**
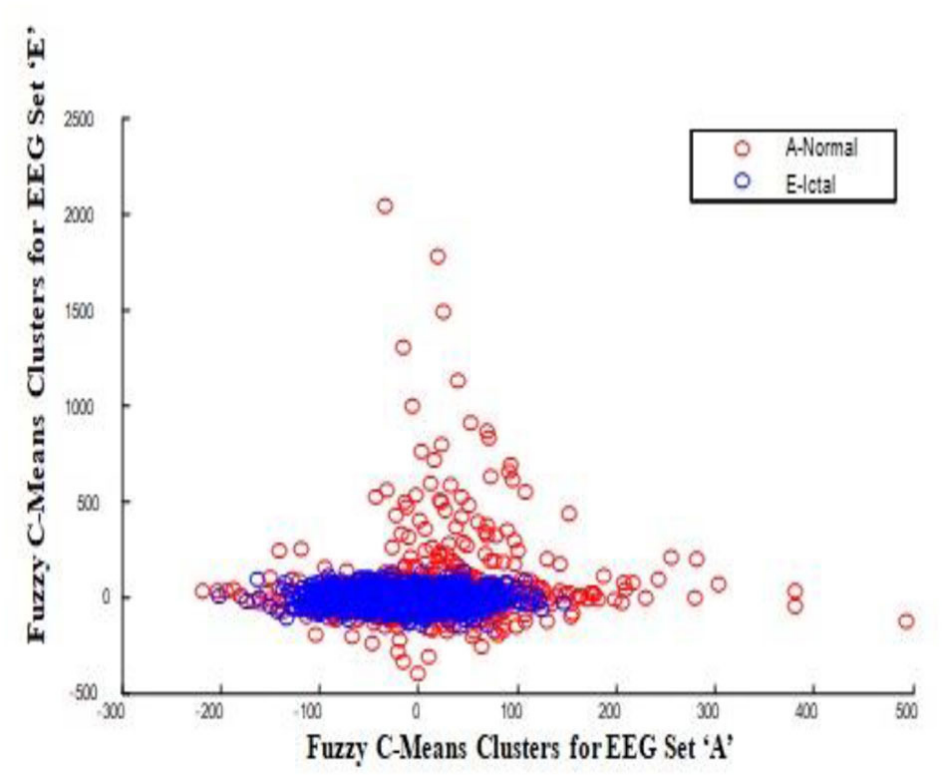
FCM clusters for EEG sets A vs. E.

**Figure 5:**
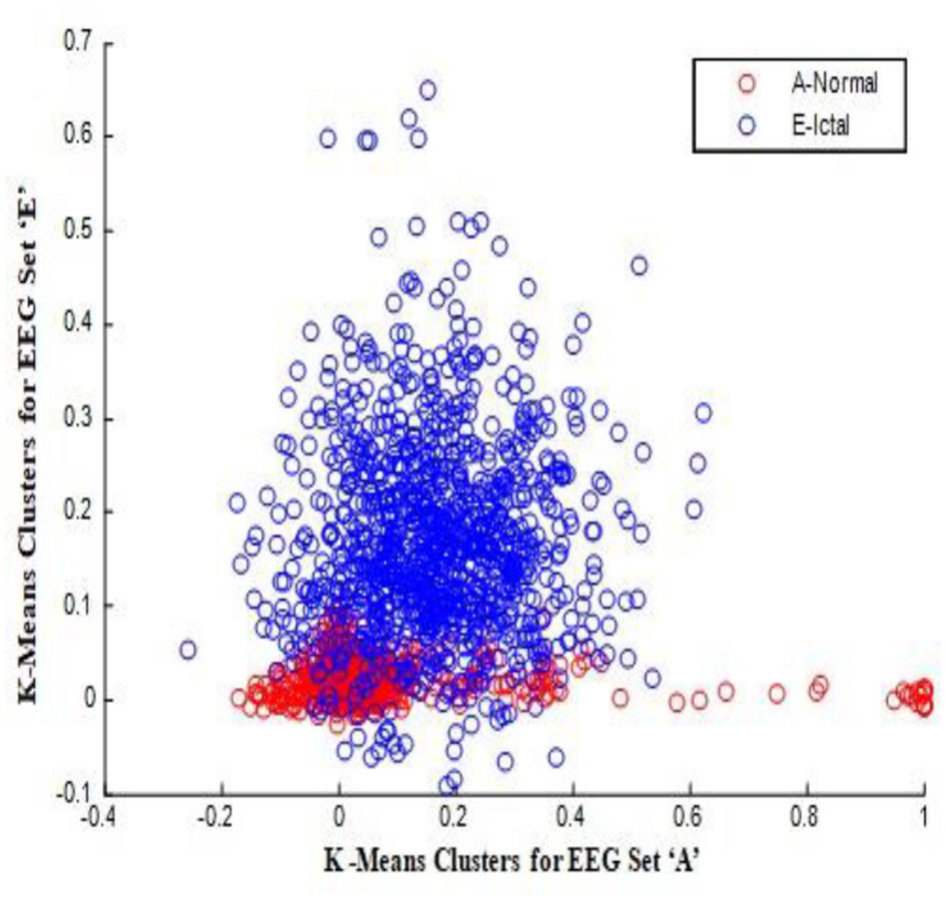
K-means clusters for EEG sets A vs. E.

**Figure 6:**
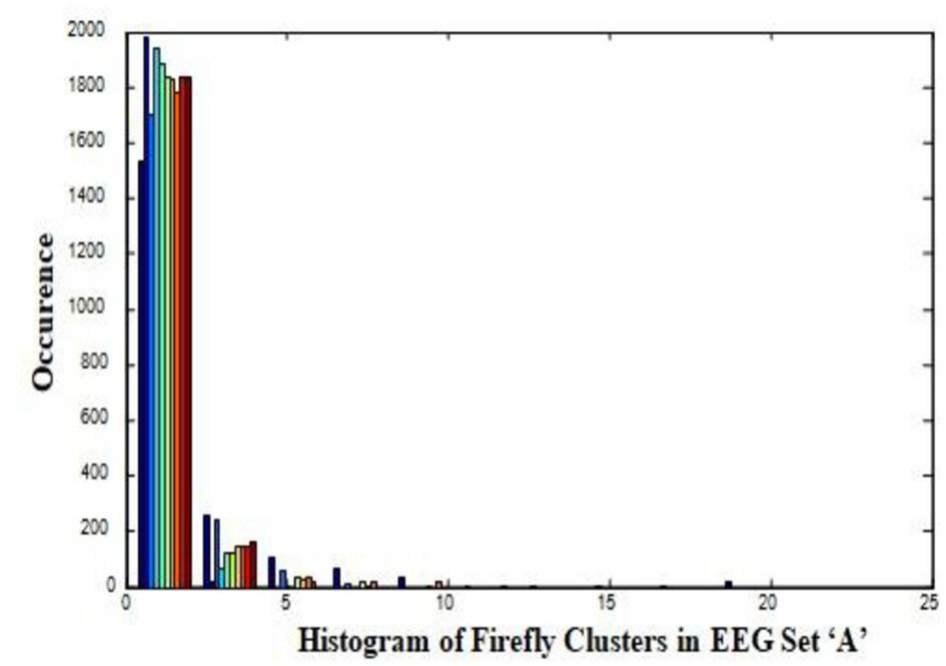
Histogram of Firefly clusters in EEG set “A”.

**Figure 7:**
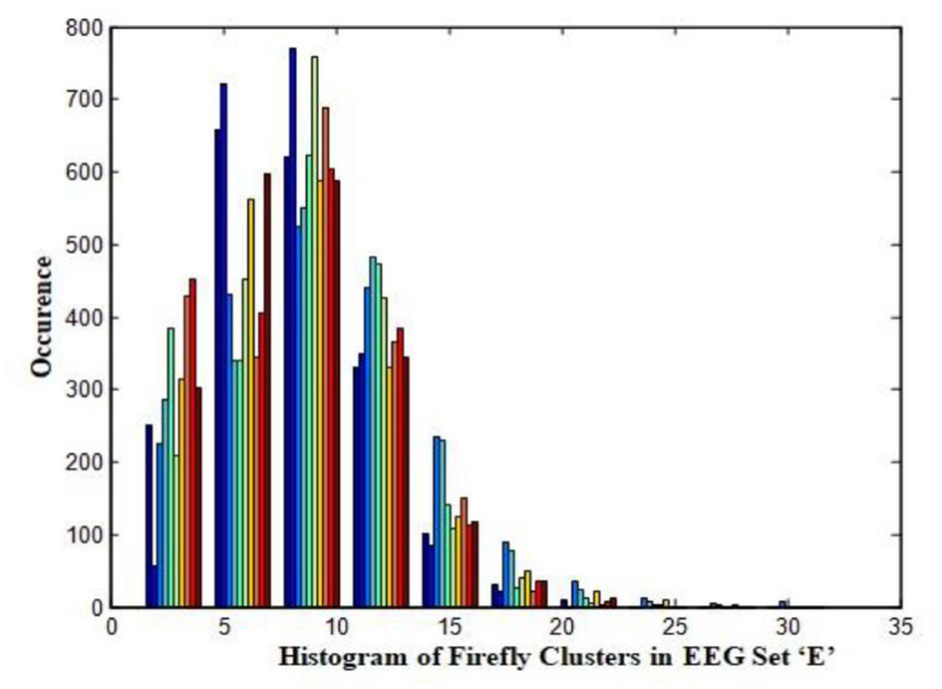
Histogram of Firefly clusters in EEG set “E”.

In this work, a total of (4,097 × 100) A-E EEG data are clustered based on the cluster center features as (4,097 × 10) A-E EEG data. Therefore, an effective data reduction of ten times is achieved. The reduced features must be analyzed for the presence of non-linearity and dynamic states of the EEG signal. It is a two-step process consisting of scattering with histogram plots and entropy extraction. Scatter plots and histograms can easily help identify the presence of non-linearity in the features. Figures 2, 3 depict the scatter plot of Dragonfly clusters and Firefly clusters for EEG set A vs. E. The shape of the cluster in the figures obtained shows the presence of non-linearity in the features. Figures 4, 5 depict the scatter plot of FCM clusters and K-means clusters for EEG A vs. E. Figure 6 shows the histogram of Firefly clusters in EEG set A. This histogram plot is a right-skewed one, which again establishes the presence of non-linearity in the feature. Figure 7 illustrates the histogram of Firefly clusters in EEG set E. Figure 7 exhibits the flatness in the histogram, which is different from the histogram of set A. The profound technique of extracting three types of entropy, e.g., Approximate Entropy (Pincus, 1991), Shannon Entropy (Shannon, 1948), and Sample Entropy (Richman and Moorman, 2000) for the clustered features of EEG data shows the intactness of residuals and variation as in the original EEG data.

Shannon Entropy extracts the uncertainty associated with a more specific event and its outlier characteristics. Approximate Entropy shows the presence of non-linear and complicated features in irregular data. Sample Entropy is obtained by the removal of self-matches in the Approximate Entropy. Sample Entropy is independent of data length and depicts consistency. The average entropies, which in turn represent the lower and upper bounds of the clusters, are presented in Table 3. The average value of entropy among the clusters in Table 3 indicates the availability of a distinct classification in the A-E EEG data set.

## 4. Classification of the clustered values

The clustered values are then classified with the help of 10 different classifiers such as ANN, KNN, Incremental LDA (I-LDA), NBC, Quadratic Discriminant Analysis (QDA), Decision Trees, and Random Forest (RF), as explained in the following subsections.

### 4.1. ANN classifier

Motivated and inspired by the basic functional ideas of biological neural networks, ANN is a simple learning-based classifier (Sezer et al., 2012). The posterior probabilities can be easily estimated by ANN, which in turn helps to establish classification rules and then perform the statistical analysis. There are various ANN parameters here, the ANN configuration uses training cycles = 800, momentum decay = 0.5, and learning rate = 0.3. The ANN architecture used in this work is (128-32-2). The learning algorithm used here is the Levenberg-Marquardt (LM) algorithm with a sigmoidal function.

### 4.2. KNN classifier

A given test sample is compared with the training samples that are similar, where the *k* parameter is a small positive and odd integer value. There are two important steps in this algorithm (Song et al., 2007). First, the *k* training samples that are closest to the invisible sample are found. Second, the common classification for the *k* samples is taken, and then the average of the values of its KNN is found out in the regression stage. Using a distance metric called normalized Euclidean distance, it can be defined. Between the two points *Z*_1_ = (*z*_11_, *z*_12_, ..., *z*_1*m*_) and *Z*_2_ = (*z*_21_, *z*_22_, ..., *z*_2*m*_), the distance is analyzed as:


(19) $$\begin{array}{l} dist\;(Z_{1},Z_{2})=\sqrt{\overset{m}{\underset{i=1}{\sum }}{\left(z_{1i}-z_{2i}\right)}^{2}} \end{array}$$

In this work, the KNN uses a value of *k* = 2, and the measure types have been selected as mixed measures, thus allowing the mixed Euclidean distance as the best option.

### 4.3. I-LDA classifier

It is a kind of LDA classification technique that can easily update and classify the cluster features through the simple observation of new samples (Chu et al., 2015). The I-LDA classifier was trained with pre-ictal and ictal feature vectors. In the training set, the sampling strategy was used to randomly balance the number of pre-ictal and ictal segments. During the testing phase, the trained classifier was tasked with categorizing the incoming epoch as a pre-ictal or an ictal state. For the pre-ictal state, the binary result of the classifier was zero, and for the ictal state, it was one. To smooth the results, a median filter was used. The prediction alarm would be raised if Σ*T*_*j*_ = α_*j*_, where *T*_*j*_ is consecutive “1 s”, with a progressive window of 1 s and α_*j*_ is a patient dependent threshold,. The value of α_*j*_ is obtained from the training data set. If the range falls within the prediction zone, then the alarm is positive; otherwise, it is considered as a false alarm.

### 4.4. NB classifier

This simple statistical probabilistic classifier was developed based on the application of Bayes' theorem (Islam et al., 2007). The naive Bayesian technique assumes that the calculation of the NBC is easier than the exponential complexity. Here, it proves its efficacy by analyzing the fact that certain features of a class are irrelevant to other features. Each feature is considered independently to calculate its respective probability, which helps to calculate the probability of a certain class, which will be the classification outcome.

### 4.5. SVM classifiers

Based on the principles of structural risk minimization and statistical learning theory, SVM classification was developed (Swami et al., 2014). The main idea of the SVM is to map the input data into a higher-dimensional space. Once mapped, an optimal separating hyperplane between the two data classes of the transformed space is determined. SVMs can easily map the inseparable data into a high-dimensional space by means of constructing a linear kernel function. When the data are not linearly separable, which is the case with nonlinear classifier models, SVMs can clearly provide a better fit of the hyperplane to the input data set. Originally designed as a two-class classifier, its application was later extended to multiclass classification. A set of pairwise classifiers is employed based on one-against-one decomposition. For the binary SVM classifier, the decision function is expressed as:


(20) $$\begin{array}{l} f\left(y\right)=sgn\;\left(\overset{s}{\underset{j=1}{\sum }}z_{j}\alpha_{j}k\left(y_{j},y\right)+w\right);\;0<\alpha_{j}<Q \end{array}$$

where *sgn* denotes a sigma function; *k*(*y*_*j*_, *y*) denotes a kernel function; and *w* denotes the bias of the training samples. Several kernel functions, such as the Linear kernel, Polynomial kernel, and Radial Basis Function (RBF) kernel, are used in this work. Between the model complexity and the training error, to control the trade-off, the regularization parameter *Q* is utilized and is calculated as follows:


(21) $$\begin{array}{l} Q=\frac{N}{\overset{N}{\underset{j=1}{\sum }}K\left(y_{j},y\right)} \end{array}$$

where *N* denotes the size of the training set.

By utilizing a set of decision functions *f*_*kh*_, a binary or multiclass classification problem arises. With the help of the following formula, the class decision is obtained as follows:


(22) $$\begin{array}{l} f_{k}\left(y\right)=\overset{n}{\underset{j=1}{\sum }}sgn\;\left(f_{kh}\left(y\right)\right) \end{array}$$

where *kh* denotes each pair of classes extracted from separate target classes, *n* denotes the number of separate target classes. Initially, a label is assigned to the class as follows:


(23) $$\begin{array}{l} \arg\max f_{k}\left(y\right),\left(k=1,2,...,n\right) \end{array}$$

The conversion from the *n*-class classification problem to the (*n*(*n*1)/2) two-class problem is generally done by pairwise classification, which helps to cover all the pairs of classes.

### 4.6. QDA classifier

Among statistics, signal processing, and pattern recognition, it is widely used to seek a quadratic combination of features that are responsible for analyzing an example into two or more types of categorizations (Heijden et al., 2005). The process of discriminating quadratic multiplication factors is used for both classification and dimensionality reduction, but in our work it has been used only for classification.

### 4.7. Decision tree classifier

Decision Trees utilize the top-down construction method to recursively split the data set into smaller subsets (Wang et al., 2012). Utilizing the concept of information entropy, a decision tree is built by the classifier for the data set. By splitting the data into a smaller number of subsets, a decision can be made for each attribute in a decision tree. The attribute that gives the highest information gain is easily evaluated by this algorithm. Once an attribute is selected, the data set is divided into further subsets. As a result of the tree structure, each inner node corresponds to input attributes, each branch indicates a range of values within that attribute, and each leaf accounts for a good classification.

### 4.8. RF classifier

To solve the classification problem, one of the successful ensemble techniques used in the machine-learning approach is RF (Mursalin et al., 2017). A collection of Decision Trees is presented here that could act as a single classifier, also enabling multi-classification models and tasks. To achieve the most stable tree classification, various subsets of the training data are fed to each tree, thereby resulting in a generalized experience for the classifier. The data set is divided into two parts. First, each tree is trained using the bootstrapping method, and the second part is utilized to assess the classification accuracy. To obtain a high-variance classifier, each tree is allowed to reach its maximum depth. The splitting process continues until the pre-defined termination condition is achieved. Once the forest is established, the total number of subsets remains a constant value. From the root node to the leaf node, the journey is made for the unlabeled instances in the classification. The determination of each class that has the maximum votes from all the decision trees is analyzed for a final decision.

## 5. Results and discussion

The effectiveness of a classifier can be evaluated with many performance evaluation formulas, such as Sensitivity, Specificity, and Accuracy, and is represented as follows:


(24) $$\begin{array}{l} Sensitivity=\frac{TP}{TP+FN} \end{array}$$

(25) $$\begin{array}{l} Specificity=\frac{TN}{TN+FP} \end{array}$$

(26) $$\begin{array}{l} Accuracy=\frac{TP+TN}{TP+TN+FP+FN} \end{array}$$

True Positive (TP) and True Negative (TN) indicate the correct classification of the number of epileptic seizures and seizure-free signals. False Positive (FP) and False Negative (FN) denote the incorrect classification of the number of epileptic seizures and seizure-free signals.

The representation of the Mean Square Error (MSE) is expressed as follows:


(27) $$\begin{array}{l} MSE=\frac{1}{Z}\overset{Z}{\underset{i=1}{\sum }}{\left(V_{i}-W_{j}\right)}^{2} \end{array}$$

where the observed value at a given instant of time is expressed as *V*_*i*_, *W*_*j*_ represents the target value at the model *j*; *j*=1 to 10, and *Z* represents the total number of significant observations per patient - in our case, 4097. The training was carried out in a regressive manner, and the MSE value of the classifiers was drastically reduced to the maximum possible extent. The training of all the classifiers was done to achieve a zero-training MSE. The selection of the hyper parameters in the classifiers is of two types; they are attaining the minimum MSE and the number of iterations with reasonable accuracy. As in the case of the classifier (SVM), the maximum number of iterations is fixed at 450 for a minimum MSE of (10^−05^), and apart from this, another constraint of obtaining continuous achievement of the minimum MSE for at least three consecutive iterations is done. In order to avoid the local minima problem, multiple runs of the algorithm are performed. Under this condition, the classifiers are trained to obtain valuable parametric values with reasonable accuracy.

To ensure the effectiveness and reliability of the classifiers, cross-validation is used. For both training and testing, the available data is split up into subsets of equal size. The first subset is chosen as the test set, and the other K-1 subsets are combined to form the training and validation sets. After training the classifier using these subsets, the classification performance of the test set is recorded. The process is then repeated so that each of the K-1 subsets acts as a test set in turn. The final classification performance is the average of the K test set results. In this paper, a value of 10 was used for K, and the experiment was repeated ten-fold.

### 5.1. Selection of KNN hyper parameters

Since the problem is of two classes in nature, that is, normal and epileptic sets of EEG signals, the K value for the KNN classifier has to be chosen as two or more than that. In order to select the hyper parameters for the KNN classifier, we identified the penalty function as Euclidean distance, the similarity index error as MSE, and the number of iterations as the cost function. As in the case of KNN classifiers, the same procedure is repeated for different values of K = 2, 4 to identify the optimal number of iterations in terms of a low MSE value. Table 4 shows the performance of MSE with a different number of iterations for KNN classifiers at different K values.

**Table 4:** Performance of MSE with different numbers of iterations for KNN classifiers at different K values.

**Number of iterations**	**MSE at different K values**	
	***K* = 2**	***K* = 4**
50	0.000475	0.000001
100	0.0004	4.00E-03
150	0.000204	7.84E-04
200	4.49E-05	0.002025
225	3.48E-05	3.60E-04
250	0.000025	0.000122
300	2.3E-05	0.0001
350	2.12E-05	0.00059
375	1.68E-05	0.000128
400	1.09E-05	0.000292
500	4.41E-06	6.25E-04
600	4E-06	0.00049
800	2.25E-06	1.00E-04
1000	1.96E-06	0.000202
1200	0.000001	5.76E-04
1400	3.6E-07	0.000302

**Figure 8:**
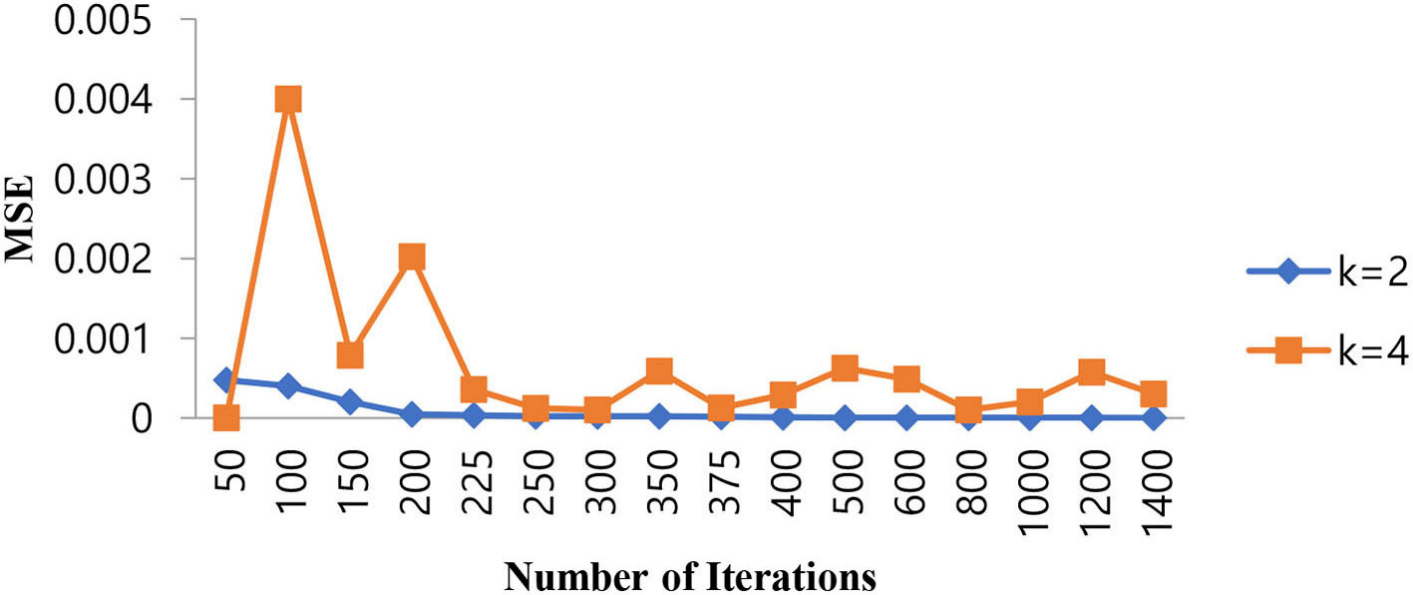
Performance of the MSE in the number of iterations for the KNN classifier at different K values.

As shown in Table 4, when the value of K is 2, the KNN classifier converges at a low number of iterations (300) at an MSE value of 2.3E-05, and any further increases in the number of iterations further decrease the MSE value. For K = 4, the KNN classifier is either impacted by local minima or has a flattened MSE effect. Therefore, for the KNN classifier, the K value is selected as 2. The same performance of the MSE is also depicted in Figure 8.

### 5.2. Selection of SVM hyper parameters

The selection of hyperparameters in an SVM classifier is done by two functions, i.e., penalty and cost functions. In this paper, we are discussing three types of SVM classifiers, namely SVM-RBF, Polynomial SVM, and Linear SVM. In the case of SVM-RBF and Polynomial SVM classifiers, the hyper parameters are selected by the grid-based search method. For the SVM-RBF classifier, the gamma value of the RBF kernel must be chosen. For this, MSE will be the penalty function, and the number of iterations will be the cost function as it attains a good gamma value for low MSE at a lower number of iterations. By varying the gamma value of the RBF kernel as (0.2, 0.4, 0.6, 0.8, 1.0, 1.2, 1.5, 2.0, and 2.2) and increasing the number of iterations in suitable steps, we calculated the MSE of the above mentioned SVM-RBF classifier. Table 5 shows the performance of the MSE in terms of the number of iterations for SVM-RBF at different gamma values.

**Table 5:** Performance of the MSE in the number of iterations for SVM-RBF at different gamma values.

**Number of iterations**	**MSE at different gamma values**								
	**G = 0.2**	**G = 0.4**	**G = 0.6**	**G = 0.8**	**G = 1.0**	**G = 1.2**	**G = 1.5**	**G = 2.0**	**G = 2.2**
50	0.00012321	0.00014884	0.00020164	7.744E-05	5.184E-05	0.000001	0.000328	1.37E-05	0.000511
100	5.476E-05	3.6E-05	9.025E-05	0.00017689	1.44E-06	0.000094249	0.000161	2.3E-05	2.81E-05
150	0.00012996	0.00011236	1.1664E-05	0.00018496	0.000051529	2.401E-05	1.96E-06	5.18E-05	8.10E-05
200	1.849E-05	7.921E-05	7.921E-05	1.849E-05	0.00002304	1.68921E-05	0.000801	2.92E-05	9.00E-05
225	7.569E-05	6.4E-05	0.000169	5.184E-05	0.00023409	0.00097969	0.002275	1.40E-07	6.40E-05
250	6.084E-05	0.000144	0.00019044	4.489E-05	0.00206116	0.000225	1.02E-05	4.00E-08	6.40E-06
300	5.929E-05	6.4E-05	0.00031684	0.000012996	0.00178929	6.724E-05	7.57E-05	3.51E-08	6.40E-06
350	0.0001	8.649E-05	0.000196	0.00011881	0.00147456	8.1E-05	0.000174	1.00E-08	7.92E-05
375	2.916E-05	0.00016129	4.096E-05	4.9E-05	1.024E-05	0.00042025	1.94E-05	5.00E-09	3.03E-05
400	4.9E-05	0.00022201	3.721E-05	0.0000169	1.66E-05	2.916E-05	0.000154	4.10E-09	8.10E-05
500	6.4E-05	0.00011025	0.000018496	0.00010609	0.000063504	2.304E-05	5.93E-05	4.00E-09	3.60E-05
600	0.00020736	6.889E-05	8.464E-05	2.704E-05	2.52004E-06	0.00018769	5.78E-05	2.70E-09	4.90E-05
800	0.00014161	1.0609E-05	6.724E-05	2.209E-05	0.00002025	0.00040401	0.000243	1.90E-09	8.41E-05
1000	6.561E-05	2.6569E-05	0.0000121	0.00022801	0.00000121	0.00037636	6.24E-05	1.60E-09	1.96E-05
1200	5.776E-05	0.000053361	2.304E-05	1.764E-05	1.7956E-06	3.31776E-05	0.000973	1.40E-09	6.40E-05
1400	7.14025E-05	2.43049E-05	4.489E-05	2.07E-05	5.9049E-06	1.47456E-05	5.76E-06	1.03E-09	6.64E-05

**Figure 9:**
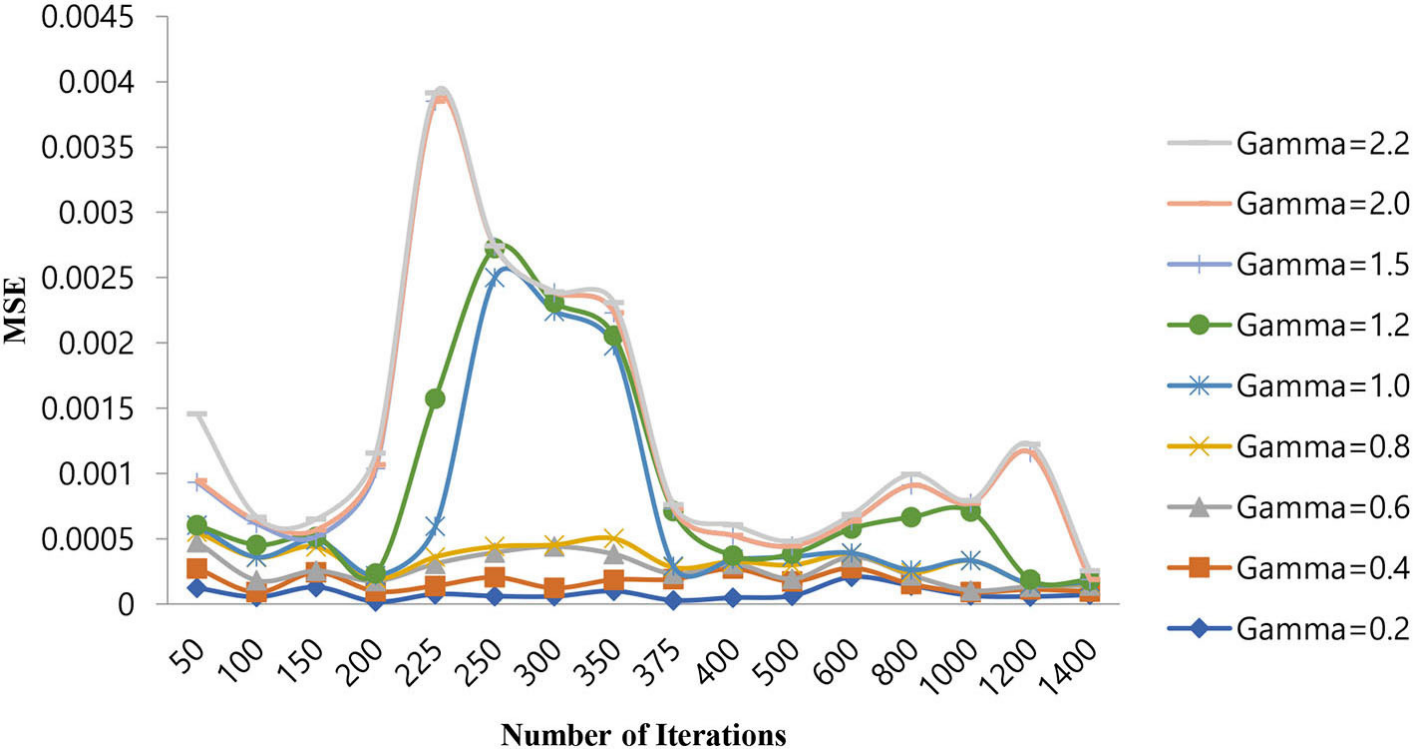
Performance of the MSE in the number of iterations for SVM-RBF at different gamma values.

From Table 5, it can be seen that for a gamma value of 2.0, a lower MSE of 4.00E-08 is obtained at a number of iterations of 250. Furthermore, in this case, an increase in the number of iterations is shown as a decrease in the MSE value. For all other gamma values, the SVM-RBF classifier is either impacted by local minima or has a flattened MSE effect. Therefore, in the case of the SVM-RBF classifier, the gamma value is selected as 2.0. The same performance of the MSE is also depicted in Figure 9.

As shown in Figure 9, for a gamma value of 2.0, a lower MSE value of 4.00E-08 is attained for a number of iterations of 250.

### 5.3. Selection of hyper parameters for the polynomial SVM classifier

The selection of hyper parameters for the Polynomial SVM classifier is performed by the grid search method, just as for the SVM-RBF classifier. The MSE is considered the penalty function, and the number of iterations is the cost function, which controls the order of the polynomial (P). Table 6 shows the performance of the MSE in terms of the number of iterations for the Polynomial SVM classifier at different polynomial orders.

**Table 6:** Performance of the MSE in terms of the number of iterations for the Polynomial SVM classifier at different polynomial orders.

**Number of iterations**	**MSE at different polynomial orders**					
	***P* = 2**	***P* = 3**	***P* = 4**	***P* = 5**	***P* = 6**	***P* = 8**
50	4.90E-06	0.000001	0.000053824	8.649E-05	0.00073984	0.00016129
100	1.44E-06	4.225E-05	0.000048841	9.409E-05	0.00061009	0.000144
150	4.9E-07	5.184E-05	0.00073984	1.00E-05	0.0007569	0.000018769
200	6.4E-07	5.184E-05	0.00003984	1.1664E-05	0.000784	8.70489E-05
225	3.6E-07	5.184E-05	4.225E-05	0.000073984	0.00047089	1.369E-05
250	2.20E-07	1.089E-05	3.96E-05	7.056E-05	0.00055696	1.024E-05
300	1.10E-07	0.000073984	6.40E-06	1.96E-05	0.00022201	2.6244E-06
350	1.51E-07	1.77E-05	7.744E-06	5.184E-05	0.00036481	0.001681
375	1.40E-07	5.776E-05	7.84E-06	2.704E-05	0.00010201	5.76E-06
400	1.90E-07	0.000026244	5.184E-06	4.90E-06	2.03E-04	2.63169E-05
500	1.18E-07	0.000073984	1.1236E-06	1.69E-06	6.5536E-05	7.29E-05
600	1.40E-07	5.184E-05	0.00000196	5.1984E-06	0.00018225	6.67489E-05
800	9E-08	1.444E-05	4.9729E-06	5.184E-05	0.00013456	1.12225E-05
1000	6.40E-08	8.836E-05	7.3984E-06	2.809E-05	0.00058564	2.304E-05
1200	3.60E-08	4.00E-06	2.401E-05	0.00000025	8.10E-05	0.000022801
1400	1.60E-08	2.61E-06	5.184E-05	4.00E-06	2.2801E-06	2.01601E-05

**Figure 10:**
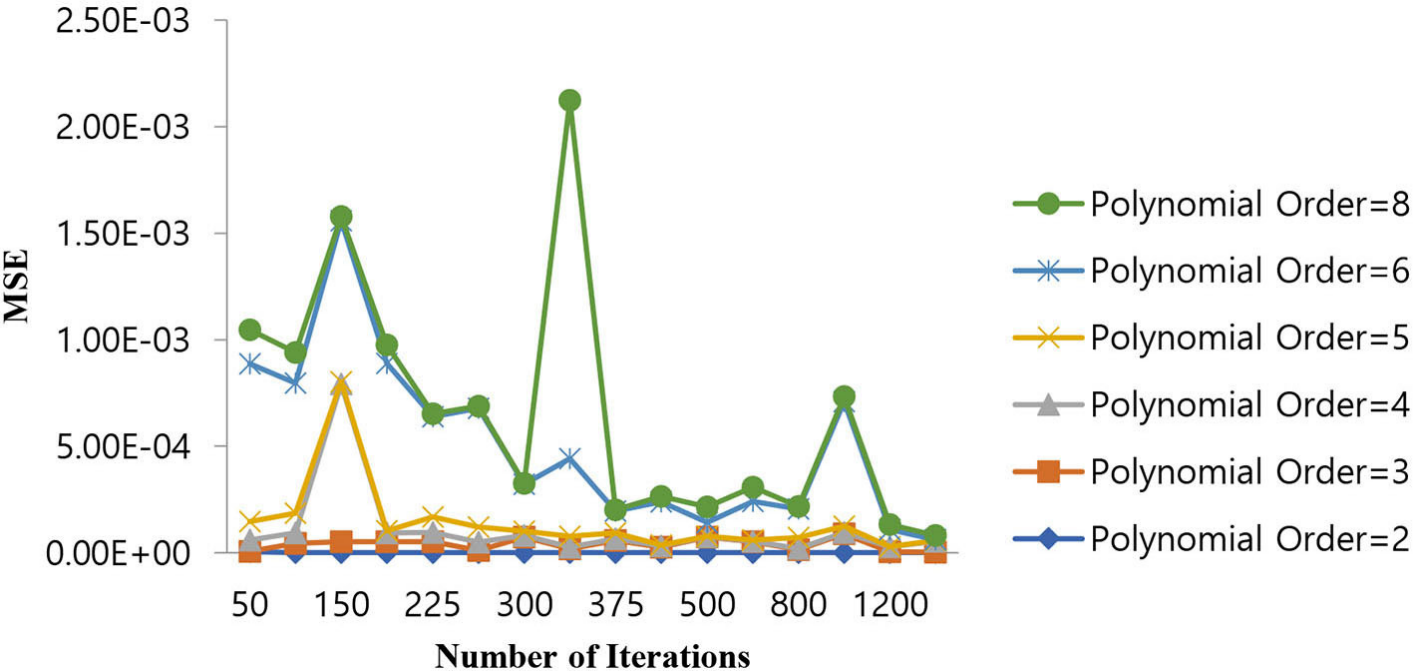
Performance of MSE in the number of iterations for the Polynomial SVM classifier at different orders.

As shown in Table 6, the polynomial of order 2 converges at a lower number of iterations (300) at an MSE value of 1.10E-07, and any further increases in the number of iterations further decrease the MSE value. For all other polynomial orders, the Polynomial SVM classifier is either impacted by local minima or has a flattened MSE effect. Therefore, for the Polynomial SVM classifier, the order is selected as 2.0. The same performance of MSE is also depicted in Figure 10.

As shown in Figure 10, for a polynomial order of 2.0, a lower MSE value of 1.10E-07 is attained for a number of iterations of 300. The MSE value increases as we increase the order of the polynomial beyond 4 and the number of iterations beyond 400.

### 5.4. Selection of hyper parameters for the SVM linear classifier

The selection of hyper parameters for the Linear SVM classifier incorporates a random search procedure. The Stochastic Gradient Decedent (SGD) algorithm was used to identify the better regression function with a low MSE along with the optimal number of iterations. The operation of the SGD algorithm is controlled by the MSE as the penalty function and the number of iterations as the cost function. Table 7 shows the performance of the MSE in terms of the number of iterations for the Linear SVM classifier for the SGD algorithm.

**Table 7:** Performance of the MSE in the number of iterations for the Linear SVM classifier for the SGD algorithm.

**Number of iterations**	**MSE**
50	2.30E-05
100	0.00061504
150	1.024E-05
200	0.000225
225	7.84E-05
250	3.31E-05
300	3.24E-05
350	6.40E-05
375	1.69E-06
400	4.97E-05
500	1.51E-04
600	2.34E-04
800	0.00021025
1,000	1.23E-06
1,200	1.32E-06
1,400	1.24E-06
1,600	2.3104E-07
2,000	1.69E-08

**Figure 11:**
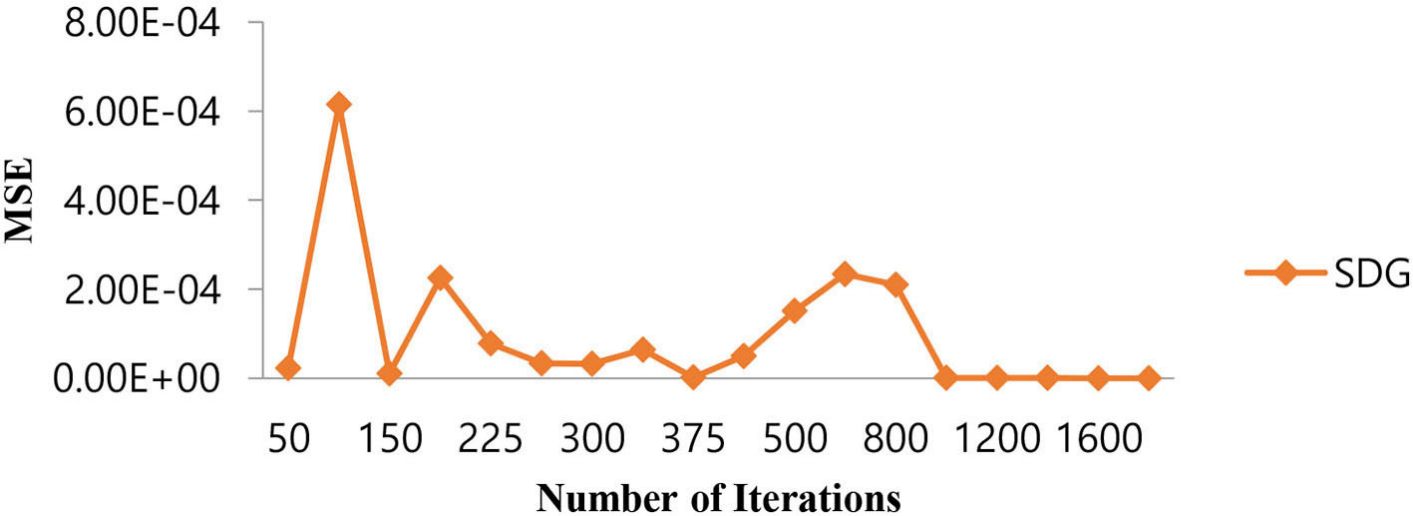
Performance of the MSE in the number of iterations for the Linear SVM classifier for the SGD algorithm.

As shown in Table 7, in the case of the SGD algorithm, the lowest MSE value is attained only after 1,000 (one thousand) iterations. Further, if we increase the number of iterations beyond 1,000, the MSE becomes a constant. Figure 11 shows the performance of the MSE in terms of the number of iterations for the Linear SVM classifier for the SGD algorithm. It is observed from Figure 11 that the number of iterations from 50 to 800 shows that the MSE value of the Linear SVM classifier follows many ups and downs and is also trapped in the local minima case.

Table 8 shows the consolidated result analysis of K-means clusters with the different classifiers. Table 9 shows the consolidated result analysis of FCM clusters with the different classifiers. Table 10 shows the consolidated result analysis of Cuckoo search clusters with the different classifiers. Table 11 shows the consolidated result analysis of Dragonfly clusters with the different classifiers. Table 12 shows the consolidated result analysis of Firefly clusters with the different classifiers. Table 13 shows the consolidated result analysis of Modified Firefly clusters with the different classifiers. Table 14 shows the consolidated MSE analysis with K-means, FCM, Cuckoo search, Dragonfly, Firefly, and Modified Firefly clusters. In this study, we used a 10-fold training and testing strategy.

**Table 8:** Consolidated result analysis of K-means clusters with the different classifiers.

**Classifiers**	**Sensitivity (%)**	**Specificity (%)**	**Accuracy (%)**
ANN	56.57656	100	78.28828
KNN	71.875	100	85.9375
I-LDA	61.46	100	80.73
NBC	100	97.92	98.96
Linear SVM	97.92	100	98.96
Polynomial SVM	94.79	100	97.395
SVM–RBF	92.71	100	96.355
QDA	91.41	100	95.705
Decision Trees	69.795	100	84.8975
RF	100	80.225	90.1125

**Table 9:** Consolidated result analysis of FCM clusters with the different classifiers.

**Classifiers**	**Sensitivity (%)**	**Specificity (%)**	**Accuracy (%)**
ANN	64.19	100	82.095
KNN	100	89.59	94.795
I-LDA	100	85.42	92.71
NBC	100	89.0675	94.53375
Linear SVM	90.11	100	95.055
Polynomial SVM	95.31	100	97.655
SVM–RBF	100	77.625	88.8125
QDA	100	96.875	98.4375
Decision Trees	97.92	100	98.96
RF	73.4375	100	86.71875

**Table 10:** Consolidated result analysis of Cuckoo search clusters with the different classifiers.

**Classifiers**	**Sensitivity (%)**	**Specificity (%)**	**Accuracy (%)**
ANN	87.5	100	93.75
K-NN	82.94813	100	91.47406
I-LDA	75	100	87.5
NBC	100	96.3525	98.17625
Linear SVM	98.96	100	99.48
Polynomial SVM	91.67	100	95.835
SVM–RBF	67.969	100	83.9845
QDA	70.05625	100	85.02813
Decision Trees	65.232	100	82.616
RF	100	90.11	95.055

**Table 11:** Consolidated result analysis of Dragonfly clusters with the different classifiers.

**Classifiers**	**Sensitivity (%)**	**Specificity (%)**	**Accuracy (%)**
ANN	80.7375	100	90.36875
KNN	51	100	75.5
I-LDA	65.884	100	82.942
NBC	62.5	100	81.25
Linear SVM	80.225	100	90.1125
Polynomial SVM	100	81.25	90.625
SVM–RBF	94.79	100	97.395
QDA	93.75	100	96.875
Decision Trees	91.15	100	95.575
RF	77.625	100	88.8125

**Table 12:** Consolidated result analysis of Firefly clusters with the different classifiers.

**Classifiers**	**Sensitivity (%)**	**Specificity (%)**	**Accuracy (%)**
ANN	100	68.75	84.375
KNN	59.38	100	79.69
I-LDA	100	83.86	91.93
NBC	51.5	100	75.75
Linear SVM	100	84.38	92.19
Polynomial SVM	66.699	100	83.3495
SVM–RBF	79.7125	100	89.85625
QDA	71.09875	100	85.54938
Decision Trees	89.59	100	94.795
RF	100	78.4125	89.20625

**Table 13:** Consolidated result analysis of modified Firefly clusters with the different classifiers.

**Classifiers**	**Sensitivity (%)**	**Specificity (%)**	**Accuracy (%)**
ANN	57.42563	100	78.71281
KNN	100	89.59	94.795
I-LDA	100	96.3525	98.17625
NBC	100	91.41	95.705
Linear SVM	95.31	100	97.655
Polynomial SVM	95.83	100	97.915
SVM–RBF	100	65.884	82.942
QDA	100	80.225	90.1125
Decision Trees	56.7725	100	78.38625
RF	71.68094	100	85.84047

**Table 14:** Consolidated MSE result analysis with K-means, FCM, Cuckoo search, Dragonfly, Firefly, and Modified Firefly clusters, with classifiers.

**Consolidated analysis**	**K-means clustering**	**FCM clustering**	**Cuckoo search clustering**	**Dragonfly clustering**	**Firefly clustering**	**Modified firefly clustering**
Classifiers	MSE	MSE	MSE	MSE	MSE	MSE
ANN	9.41E-05	5.04E-05	4E-06	1.37E-05	3.6E-05	7.92E-05
KNN	2.5E-05	2.25E-06	9.61E-06	0.000529	6.89E-05	2.25E-06
I-LDA	6.08E-05	6.25E-06	2.02E-05	4.62E-05	8.41E-06	1.6E-07
NBC	4E-08	2.89E-06	1.6E-07	5.62E-05	0.0004	1.21E-06
Linear SVM	4E-08	1.96E-06	1E-08	1.44E-05	7.84E-06	3.6E-07
Polynomial SVM	4.9E-07	3.6E-07	0.000001	1.23E-05	4.36E-05	2.5E-07
SVM–RBF	8.1E-07	1.76E-05	3.97E-05	4.9E-07	1.52E-05	4.62E-05
QDA	1.21E-06	9E-08	3.25E-05	6.4E-07	2.92E-05	1.44E-05
Decision Trees	3.36E-05	4E-08	4.76E-05	1.44E-06	2.25E-06	9.02E-05
RF	1.44E-05	2.21E-05	1.96E-06	1.76E-05	1.68E-05	2.6E-05

The result of the analysis of K-means clusters when treated with the different classification techniques shows a high classification accuracy of 98.96% when NBC and Linear SVM are utilized. A low classification accuracy of 78.28% is obtained when ANN is used because of the high false alarm rate. When the K-means clusters are analyzed with the classification techniques, then the performance of the NBC and RF classifiers is impacted by the missed classification.

The result of the analysis of FCM clusters when treated with the different classification schemes shows a high classification accuracy of 98.96% when classified with Decision Trees and a high classification accuracy of 98.43% when classified with QDA. A low classification accuracy of 82.095% is obtained with a high false alarm rate of 35.8% when implemented with ANN.

The result of the analysis of Cuckoo search clusters when treated with the different classification techniques shows a high classification accuracy of 99.48% when classified with Linear SVM, while 98.17% is obtained when NBC is utilized as a classification technique. A low classification accuracy of 82.61% is obtained when Decision Trees are implemented with Cuckoo clusters.

The result of the analysis of Dragonfly clusters when treated with the different classification techniques reports a classification accuracy of 97.39% when utilized with the SVM-RBF kernel. A low classification accuracy of 75.5% is obtained when the KNN classifier is implemented.

The result of the analysis of Firefly clusters when treated with the different classification techniques reports a classification accuracy of 94.79% when classified with Decision Trees. A low classification accuracy of 75.75% is obtained when it is classified with NBC.

The result of the analysis of Modified Firefly clusters when treated with the different classification techniques reports a high classification accuracy of 98.17% when classified with I-LDA, and a classification accuracy of 97.91% is obtained when classified with Polynomial SVM. A low classification accuracy of 78.38% is obtained when classified with Decision Trees.

### 5.5. Comparison with other works

For the classification problem A vs. E, the most important works are compared with our study and tabulated in Table 15.

**Table 15:** Performance comparison with other prominent works for the A vs. E classification problem.

**Year**	**References**	**Technique**	**Classification accuracy**
2022	Prabhakar and Lee (2022a)	Sparse autoencoder with Swarm-based deep learning and reinforcement-based Q-learning	98.55%
2022	Prabhakar and Lee (2022c)	Sparse modeling with an ensemble and nature-inclined classification	98.15%
2020	Zhao et al. (2020)	End-to-end deep learning model	99.52%
2019	Raghu et al. (2019)	Matrix determinant analysis with MLP	99.45%
2018	Sharma et al. (2018)	Orthogonal wavelet analysis with Linear SVM	100%
2017	Diykh et al. (2017)	Weighted complex networks with SVM	100%
2016	Bhardwaj et al. (2016)	Empirical decomposition analysis with soft computing	98.64%
2015	Samiee et al. (2015)	Fourier transform analysis with neural networks	99.80%
2014	Kaya et al. (2014)	Binary patterns generation with Bayesian networks	99%
Proposed performance comparison analysis of clustering with machine learning (2023)	Prabhakar and Won	Clustering through Cuckoo search with Linear SVM	99.48%
Proposed performance comparison analysis of clustering with machine learning (2023)	Prabhakar and Won	a) Clustering through K-means and classifying with NBC and Linear SVM b) FCM clustering and classifying with Decision Trees	98.96%
Proposed performance comparison analysis of clustering with machine learning (2023)	Prabhakar and Won	FCM clustering and classifying with QDA	98.43%

Even though the proposed work produces slightly lower classification accuracy than the previous study, the main intention of the present study is to show that the combination of clustering with machine learning can prove to be well-suited for the classification of EEG signals for analyzing neurological disorders.

## 6. Conclusions and future work

Utilizing an EEG to detect epileptic seizures is quite a challenging task that demands a very high level of skill from doctors. The advent of computer-aided detection is a great asset to physicians for the interpretation of EEGs. This study used the concept of feature extraction through various clustering methodologies and then categorized them with the help of suitable post-classifiers. The process is simple and easy to implement. Comparatively higher classification accuracy of 99.48% was achieved when Cuckoo search clusters were utilized with Linear SVM for epilepsy detection. The second highest classification accuracy of 98.96% was obtained when K-Means clusters were utilized with NBC and Linear SVM classifiers. FCM clusters, used with a Decision Trees classifier, also gave the same classification accuracy of 98.96%. The third-best classification accuracy of 98.43% was obtained when FCM clusters were classified with QDA. The lowest classification accuracy, at 75.5%, was obtained when Dragonfly clusters were used with the KNN classifier, and the second lowest classification accuracy, at 75.75%, was obtained when Firefly clusters were classified with NBC. Future works intend to work with various other clustering techniques, such as the Whale optimization algorithm, the Moth Flame optimization algorithm, the Artificial Algae optimization algorithm, the Genetic Bee optimization algorithm, the Gray Wolf optimization algorithm, and the Fish Swarm optimization algorithm, along with different pattern recognition techniques and advanced machine learning methodologies, for effective classification of epileptic seizures in other combinations of the Bonn data set. Future works also aim to incorporate the concept of clustering and machine learning for the efficient classification of other neurological disorders. Future works, finally, aim to include a variety of transfer and deep learning techniques for the efficient classification of epilepsy from EEG signals.

## Data availability statement

Publicly available datasets were analyzed in this study. This data can be found here: https://www.upf.edu/web/ntsa/downloads/-/asset_publisher.

## Author contributions

SP: concept, methods, implementation, visualization, and writing draft. D-OW: visualization, critical analysis, revision, supervision, and funding acquisition. Both authors contributed to the article and approved the submitted version.
